# Dynamics of Multiple Trafficking Behaviors of Individual Synaptic Vesicles Revealed by Quantum-Dot Based Presynaptic Probe

**DOI:** 10.1371/journal.pone.0038045

**Published:** 2012-05-29

**Authors:** Suho Lee, Kyung Jin Jung, Hyun Suk Jung, Sunghoe Chang

**Affiliations:** 1 Department of Physiology and Biomedical Sciences, Seoul National University College of Medicine, Seoul, South Korea; 2 Neuroscience Research Institute, Medical Research Center, Seoul National University College of Medicine, Seoul, South Korea; 3 Biomembrane Plasticity Research Center, Seoul National University College of Medicine, Seoul, South Korea; 4 Bio-Max Institute, Seoul National University College of Medicine, Seoul, South Korea; 5 Department of Life Science, Gwangju Institute of Science and Technology, Gwangju, South Korea; 6 Division of Electron Microscopic research, Korea Basic Science Institute, Daejeon, South Korea; University of California, Berkeley, United States of America

## Abstract

Although quantum dots (QDs) have provided invaluable information regarding the diffusive behaviors of postsynaptic receptors, their application in presynaptic terminals has been rather limited. In addition, the diffraction-limited nature of the presynaptic bouton has hampered detailed analyses of the behaviors of synaptic vesicles (SVs) at synapses. Here, we created a quantum-dot based presynaptic probe and characterized the dynamic behaviors of individual SVs. As previously reported, the SVs exhibited multiple exchanges between neighboring boutons. Actin disruption induced a dramatic decrease in the diffusive behaviors of SVs at synapses while microtubule disruption only reduced extrasynaptic mobility. Glycine-induced synaptic potentiation produced significant increases in synaptic and inter-boutonal trafficking of SVs, which were NMDA receptor- and actin-dependent while NMDA-induced synaptic depression decreased the mobility of the SVs at synapses. Together, our results show that sPH-AP-QD revealed previously unobserved trafficking properties of SVs around synapses, and the dynamic modulation of SV mobility could regulate presynaptic efficacy during synaptic activity.

## Introduction

The presynaptic efficacy at any given synapse is defined by the size of the recycling pool with the intrinsic release probability of the SVs. Contrary to conventional models of neurotransmission [1], [2], a growing body of evidence shows that a substantial proportion of the SVs is shared between boutons [3], [4]. Such a dynamic sharing of SVs may resize the SV pools and affect presynaptic efficacy during various synaptic activities. Although intriguing, most of the previous studies about vesicle sharing comes from the analysis of the bulk-movement of SV clusters and thus, have only proven the existence of vesicle sharing between boutons; however, because of a lack of suitable techniques, they failed to provide any information regarding the trafficking behaviors or dynamic nature of a single SV in and out of synapses, and even more important, how the trafficking behaviors of a single SV around synaptic areas are affected by synaptic activity.

Recently, quantum dots (QDs) have provided invaluable information regarding the behaviors of postsynaptic receptors at the peri-active zone of synapses [5]–[9]. Since the presynaptic and postsynaptic cells in chemical synapses are separated by the synaptic cleft which is 20∼30 nm in size [10], one of the concerns was the size of a conventional QD-conjugate (>50 nm) [6]. Although such a large complex apparently labels postsynaptic receptors in the peri-active zone, for application to a presynaptic vesicle that is ∼30 nm in diameter, a smaller QD-conjugate is required. Recently, QDs without conjugation have been used to detect the kiss-and-run event of SV fusion at presynaptic boutons [11], but these untethered QDs are not applicable to continuous monitoring of SV mobility.

In this study, to continuously monitor the trafficking behaviors of presynaptic SVs at synapses, a QD based presynaptic probe was created, and using the single-particle QD tracking technique, various parameters underlying the multiple diffusive behaviors and dynamics of individual SVs were measured. Our findings not only support the ideas of the existence of extrasynaptic mobility and the substantial cross-communication of SVs between synapses, but also provide novel evidence in that SV mobility around synapses is dynamically modulated by synaptic activities that could involve the cytoskeletons. Such a modulation of the SV diffusion parameters regulates the presynaptic performance, which underlies the presynaptic efficacy and subsequently certain forms of synaptic plasticity.

## Results and Discussion

Unlike QD-based tracking of postsynaptic receptors, for application to presynaptic vesicles, the QD-conjugate should not only penetrate the synaptic cleft (∼30 nm) but also be encapsulated in a SV of ∼30 nm in diameter. Our labeling method involved the luminal domain of the synaptoPHluorin (sPH; the fusion protein of VAMP2 and the ecliptic GFP) fused to a 15-amino acid acceptor peptide (AP) tag that is biotinylated by the ER-retained biotin ligase and subsequently labeled with streptavidin conjugated QDs 605 (QDs hereafter) [12].

**Figure 1:**
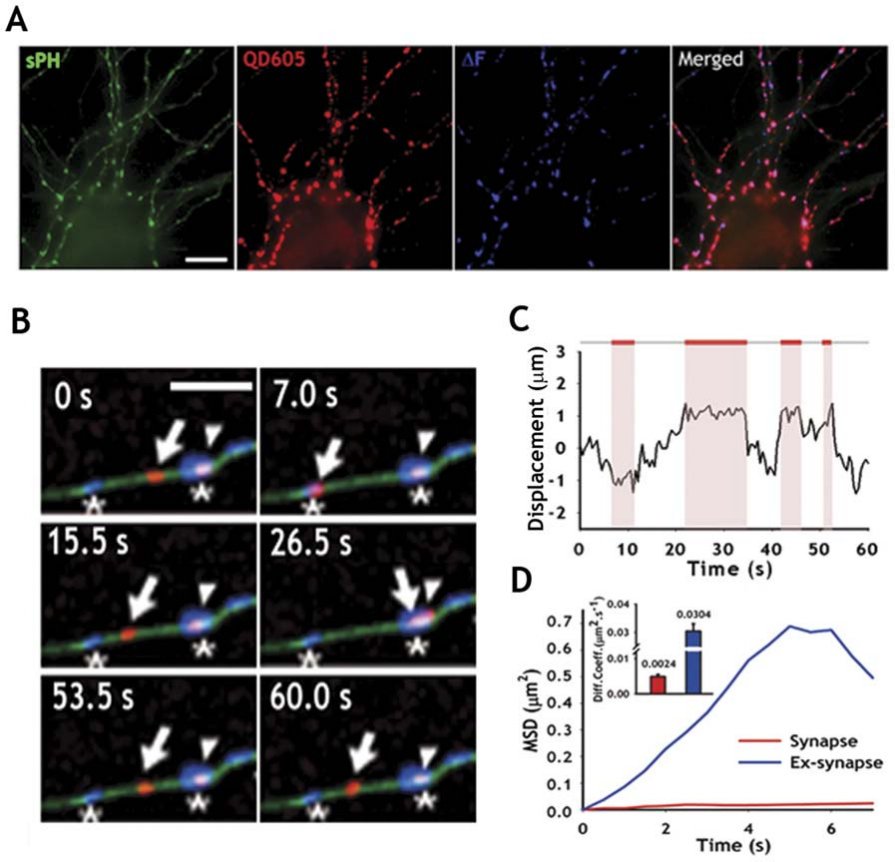
Single QD tracking of synaptic vesicles using sPH-AP-QDs. (**A**) Hippocampal neurons were co-transfected with sPH-AP and Bir-ER at DIV 12, and labeled with 1 nM streptavidin-conjugated-QD at DIV17. Blue color: the functional presynaptic terminals were identified by sPH fluorescence change (ΔF). Scale bar, 20 µm. (**B**) Example of sPH-AP-QDs (red) trafficking along the axon (green). Arrows: sPH-AP-QDs trafficking between synaptic and extrasynaptic compartments, arrowheads: sPH-AP-QDs at presynaptic terminals. asterisks: functional presynaptic terminals (blue). Scale Bar, 2.5 µm. (**C**) Instantaneous displacement change of the moving sPH-AP-QD marked by the arrow in (**B**) from its initial location (displacement = 0) along the axon during recording sequence. The *x* and *y* coordinates of QD trajectory at each time point in time-lapse images were obtained using MetaMorph track object function and the displacement from the origin to the QD trajectory at each time point was calculated and plotted. The graph parallel to *y* axis means no movement. The upper lines denote the frames in which the sPH-AP-QD is at extrasynaptic areas (gray) and synapses (red and shaded areas). (**D**) MSD versus time, calculated for a continuous sequence of images, which show the synaptic motion (red) and extrasynaptic motion (blue). Inset represents average diffusion coefficient of sPH-AP-QDs at synapses (red) and at extrasynapses (blue).

sPH-AP transfected neurons at DIV 12 were incubated with unlabeled streptavidin for 5 min on ice to block surface-resident sPH-AP and were stimulated in high KCl for 3 min in the presence of 1 nM of QDs. We found that more than 1 nM of QDs resulted in non-specific labeling. sPH is the VAMP2 protein fused to pHluorin, a modified GFP with high pH sensitivity [13]; thus, it reliably reports active presynaptic boutons by changing its fluorescence. By correlating the sPH fluorescence signal to the QD signal, we are assured that the QDs were internalized into actively recycling boutons (Fig. 1A). Compared to untethered QDs used to detect a single-round of SV fusion [11], our tethered QD-presynaptic probes were not released by the subsequent SV fusion; thus, continuous monitoring of SV dynamics was possible.

The size of the our QD-conjugate was found to be less than ∼20 nm (Fig. S1C) [14]. The interpeak spacing of the QD signal in the labeled neurons (12.46 a.u.) closely matched the unitary QD signal in agarose determined by the blinking, a hallmark of a single QD (14.03 a.u.) [15] (Fig. S1A, B), indicating that a single SV contained a single sPH-AP-QD, which was further confirmed by transmission electron microscopy (Fig. S1D).

The vesicular nature of the QD signals was confirmed by ammonium chloride treatment. Although variable batch to batch, the QDs we used showed a fluorescence increase by 15% when the pH was raised from 5.5 to 7.4 (Fig. S2A–C). To further make sure that we actually followed the QDs in the SVs and not on the surface, we measured the diffusion coefficients of the QDs on the surface, expecting that the diffusion coefficient for QDs on the surface would be different from those in the SVs. To label QDs on the surface, we preincubated the neurons with 1 µM of TTX for 20 min, followed by incubation of 20 pM of QDs in TTX-Tyrode's solution for 5 min on ice. After washing, neurons were imaged in the TTX-Tyrode's solution. We found that the surface labeling protocol resulted in the QDs having diffusion coefficients of 0.00784±0.00028 at synapses and 0.0584±0.0009 at extrasynapses. These values are significantly faster than those of the QDs labeled using our labeling protocols (0.00275±0.00069 at synapses; 0.0344±0.0058 at extrasynapses, Table 1). Moreover, our labeling protocol using 1 nM of QDs resulted in >70% of a low diffusion coefficient population (*D_coeff_* = 0.00287±0.00032, which is supposedly in the SV) and <30% of a high diffusion coefficient population at synapses (*D_coeff_* = 0.00837±0.00124, which is supposedly on the surface; Fig. S2D) as well as at extrasynapses (74.5% of *D_coeff_* = 0.0289±0.0662 and 26.4% of *D_coeff_* = 0.0602±0.00186). These values were in good agreement with the values for the surface-resident sPH found in previous studies [13] as well as in the current study using NH_4_Cl treatment (Fig. S2C). Therefore, by measuring the diffusion coefficient of the QDs, we were sure that the QDs were not on the surface but in the SVs.

**Table 1:** The values of various diffusive behaviors of sPH-AP-QDs analyzed in this study.

		Mobile fraction	D_synapse_	D_ex-synapse_	Influx frequency	Dwell time	Confinement area
**Actin**	Control	0.2201±0.0248	0.00275±0.00069	0.0344±0.00581	3.0119±0.6629	11.6±1.6724	0.1435±0.0464
	Cytochalasin B	0.1445±0.0215 *	0.00079±0.00021 *	0.0304±0.00804	1.39±0.4984 *	28.85±3.9198 *	0.0203±0.0284 *
**Microtubule**	Control	0.2478±0.0316	0.00274±0.00069	0.0304±0.00806	2.7392±0.5819	10.619±1.5864	0.1964±0.0537
	Nocodazole	0.2375±0.0198	0.00262±0.00061	0.0134±0.00381 *	3.0092±0.5279	10.9048±1.1168	0.1826±0.0451
**Gly-SP**	Control	0.188±0.016	0.00195±0.00068	0.0306±0.00552	2.6668±0.5848	11.3158±1.1574	0.170±0.0548
	Gly	0.2398±0.0202 *	0.00601±0.00078 *	0.0326±0.00535	4.2916±0.725 *	7.3684±0.9435 *	0.418±0.0901 *
	Control	0.1935±0.0136	0.00246±0.00055	0.0312±0.00355	2.7942±0.8686	10.4118±1.3934	0.166±0.052
	Gly + APV	0.1865±0.0119	0.00236±0.00045	0.0315±0.00409	2.9773±0.6615	9.8824±1.2304	0.174±0.0463
**NMDA-SD**	Control	0.1651±0.0267	0.00228±0.00052	0.0337±0.00568	2.7856±0.6207	12.0256±1.7201	0.141±0.0336
	Gly + NMDA	0.171±0.028	0.00176±0.00037 *	0.0326±0.00512	2.6344±0.4852	11.0403±1.5619	0.0833±0.0158 *

Actin, actin cytoskeleton disrupted; Microtubule, microtubule disrupted; Gly-SP, Glycine-induced synaptic potentiation; NMDA-SD, NMDA-induced synaptic depression; D_synapse_, diffusion coefficient at synapses; D_ex-synapse_, diffusion coefficient at extrasynapses; All values are mean ± s.e. *p<0.01, paired *t*-test.

We next tested whether QD labeling affects exo-endocytic trafficking of the SVs. We assumed that the total recycling pool size was ∼100 vesicles and since we knew the fluorescence intensity of a single QD (Fig. S1A, B), we estimated about 12–15 vesicles were labeled with QDs using our labeling protocol, which is ∼15% of the total recycling pool size. We measured the fluorescence changes in sPH during and after stimulation, and found that the uprising exocytotic kinetics (Fig. S3B) and post-stimulus endocytic kinetics of sPH (Fig. S3A) were not different from those of the control, indicating that our QD labeling did not affect the normal physiology of the exo-endocytic trafficking of the SVs (See also Discussion S1).

For the rest of the experiments, we labeled neurons with 100 pM of QDs and perfused Tyrode's solution for 10 min to wash out unbound QDs and to stabilize the activated neurons. This approach successfully labeled very few SVs with QDs, which allowed us to track down a single vesicle. We analyzed sPH-AP-QD lateral diffusion with the single-particle tracking technique [5]. sPH-AP-QDs displayed diffusive behaviors at the extrasynaptic compartments but remained stable for a long period when they encountered synaptic compartments (Fig. 1B). We plotted the mean square displacement (MSD) as a function of time (MSD(*nτ*)), which provides information on the diffusive behavior and diffusion coefficients [16]. The MSD (*nτ*) for the sPH-AP-QDs was linear in the extrasynaptic domains indicating free diffusion-like movement while negatively deflected in the synaptic compartments indicating confined-like movement. The average diffusion coefficient over time indicates that the extrasynaptic QDs were more mobile than the synaptic QDs (Fig. 1C, D).

**Figure 2:**
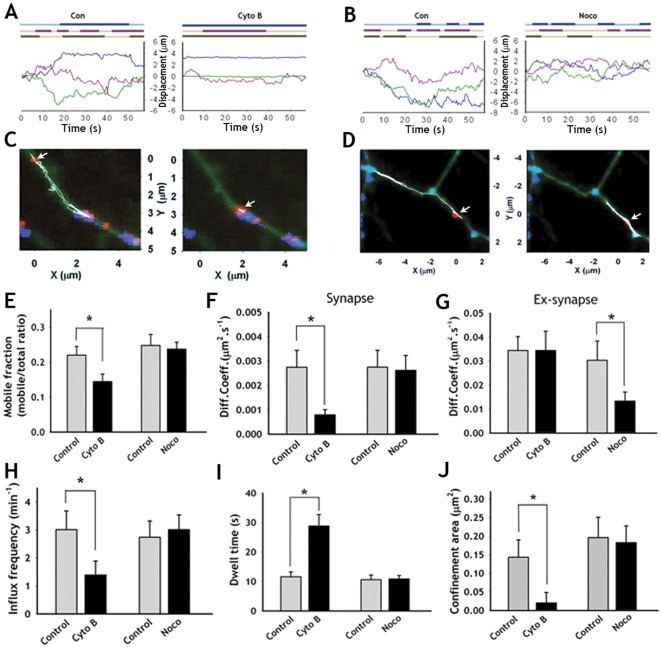
Effects of F-actin or microtubule on the diffusive behaviors of sPH-AP-QDs at synapses and extrasynaptic compartments. (**A–B**) Three representative sPH-AP-QDs displacement traces showing movement around their initial locations along the axon before and after cytochalasin B (**A**), or nocodazole (**B**) treatment. The upper colored bold line denotes the frames on which the sPH-AP-QDs are at synapses. (**C–D**) Reconstruction of a sPH-AP-QD in (**A**) or (**B**) mass center trajectories (white) for 60 s over the sPH fluorescence positive synapses (blue) images along the axons (green) before (left panel) and after (right panel) 20 min treatment with Cyto B (**C**) or Noco (**D**). The initial locations of sPH-AP-QDs were indicated by arrow. (**E**) Comparison of mobile fraction before and after Cyto B or Noco treatment. (**F–G**) Comparison of average diffusion coefficients at synapses (**F**) and at extrasynapses (**G**) before and after Cyto B or Noco treatment. (**H–J**) Comparison of influx frequency (**H**), dwell time (**I**), confinement area at synapses (**J**) before and after Cyto B or Noco treatment. Values are mean ± s.e. *p<0.01, paired *t*-test. (n = 11 neurons for Cyto B; n = 9 neurons for Noco, each neuron with at least 10 sPH-AP-QDs).

Presynaptic terminals are actin rich compartments and the SV mobility is predicted to be confined by the cytoskeleton [17]. We, however, found that actin disruption by cytochalasin B (Cyto B; 3 µg/ml) decreased the ratio of the mobile/immobile fraction significantly (Fig. 2A, C, E). This was accompanied by a 3.3-fold decrease in the diffusion coefficient at synapses (Fig. 2F, Fig. S6A and Table 1). The influx frequency (the number of entries of sPH-AP-QDs into the synapse per minute) was significantly decreased (Fig. 2A, C, H, Table 1). Cytochalasin B treatment also increased the mean dwell time (the duration that sPH-AP-QD stays at the synapse once it has entered) and decreased the confinement area (see Method) at synapses (Fig. 2I and Table 1) while it did not affect the diffusion coefficient at extrasynaptic areas (Fig. 2G).

In contrast, nocodazole treatment affected neither the diffusion coefficient nor the confinement area at synapses but induced a significant decrease in the diffusion coefficient at extrasynapses (Fig. 2B, D, F, G, Fig. S4A–D, Fig. S6B and Table 1). The influx frequency or the dwell time at synapses was not affected by nocodazole treatment (Fig. 2H, I and Fig. S4E, F and Table 1). These results suggest that, although the movement of the SVs exhibits diffusive characteristics, a substantial fraction of SV trafficking at the synaptic and extrasynaptic areas may depend on actin and microtubules, respectively (See also Discussion S1).

**Figure 3:**
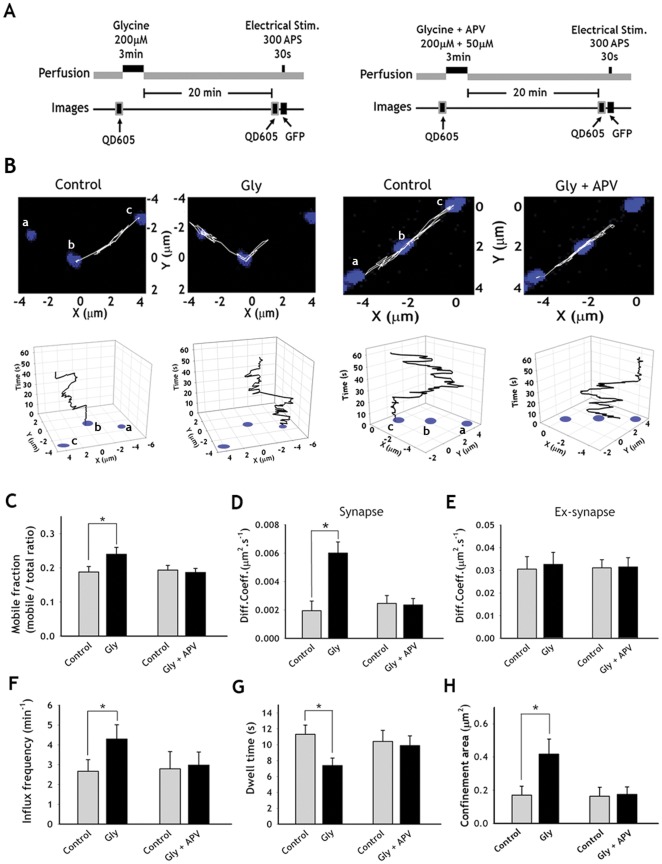
Effects of Gly-SP on the diffusive behaviors of SVs. (**A**) Schematic diagrams of Gly-SP induction and imaging protocols with (right) or without (left) APV. Hippocampal neurons labeled with sPH-AP-QDs were placed in the Mg^2+^-free extracellular solution (110 mM NaCl, 2 mM CaCl_2_, 5 mM KCl, 10 mM HPES, 30 mM Glucose, 0.5 µM TTX, 1 µM Strychnine and 20 µM Bicuculine methiodide), and applied with 200 µM glycine (with or without 50 µM APV) for 3 min to induce Gly-SP. After 20 min in the extracellular solution, sPH-AP-QDs were tracked. Electrical stimulation (300 APs at 10 Hz) was given to verify the locations of functional synapses by observing sPH fluorescence change. (**B**) Reconstruction of a sPH-AP-QD trajectories (white) for 60 s over the functional presynaptic terminals (blue) images along the axons before (left panel) and after (right panel) induction of Gly-SP in the presence (Gly+APV) or absence of APV(Gly). Lower panels: sPH-AP-QD traces over the time in 3-dimensional space. Presynaptic terminals labeled as *a*, *b*, and *c* corresponds to those in upper panel, respectively. Graphs were rotated for better representation of SV movement over the time. (**C**) Comparison of mobile fraction before (Control) and after Gly-SP (Gly) in the presence or absence of APV (Gly+APV). (**D, E**) Comparison of average diffusion coefficients at synapses (**D**) and at extrasynapses (**E**) after Gly-Sp in the presence or absence of APV. (**F–H**) Increased influx frequency (**F**), dwell time (**G**), confinement area (**H**) after Gly-SP compared with control conditions. Values are mean ± s.e. *p<0.01, paired *t*-test. (n = 11 neurons for Gly; n = 10 neurons for Gly+APV, each neuron with at least 10 sPH-AP-QDs).

Evidence for changes in presynaptic functions during synaptic activity were suggested by a number of studies [18]–[21]. Using our probe, we tested whether changes in the synaptic activity would modulate the trafficking behaviors of individual SVs. After challenging with glycine-induced synaptic potentiation (Gly-SP) protocol (Fig. 3A) [22], the rate of FM 4-64 destaining, which is known to be a reliable indicator for presynaptic release probability [21], [23], was accelerated, indicating that the presynaptic functions were enhanced by the Gly-SP(Fig. S5).

By Gly-SP induction, the diffusion coefficient at synapses, but not at extrasynapses , was dramatically increased (Fig. 3B, C, D, Fig. S6C and Table 1). The confinement area at synapses was also significantly increased, indicating that the SVs more vigorously moved around in a larger area within the synapse (Fig. 3G and Table 1). The ratio of the mobile/immobile fraction of SVs increased significantly. This was accompanied by a large increase in the influx frequency and decrease in the mean dwell time of the SVs at synapses (Fig. 3E, F and Table 1), suggesting more vesicles entered into the synapse from the extrasynaptic areas. When Gly-SP induction was blocked by NMDA receptor antagonist 2-amino-5-phosphonovaleric acid (APV), no changes in the diffusion dynamics of the SVs were observed (Fig. 3), suggesting that Gly-SP was induced postsynaptically but expressed at least in part presynaptically, which is consistent with previous reports [21], [24], [25].

**Figure 4:**
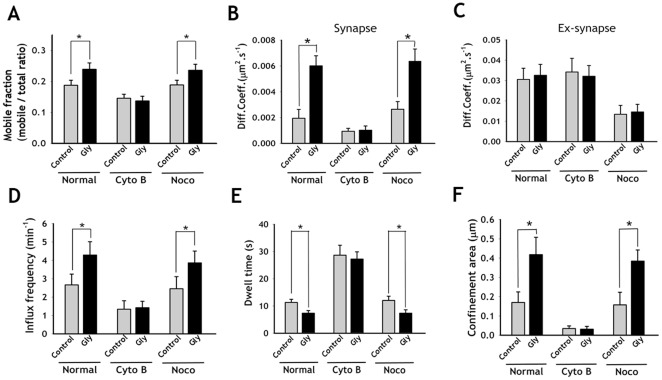
Disruption of actin but not microtubule affect Gly-SP induced changes in the SVs dynamics. After SVs labeling with streptavidin-QDs, hippocampal neurons were treated with Cyto B or Noco for 20 min to disrupt actin or microtubule. Then neurons were placed in Mg^2+^-free extracellular solution and Gly- SP was induced. (**A**) Comparison of mobile fraction before (control) and after Gly- SP (Gly) under normal (Normal), actin (Cyto B) or microtubule (Noco) disrupted condition. (**B, C**) Comparison of average diffusion coefficients at synapses (**B**) and at extrasynapses (**C**) after Gly-SP under normal, actin or microtubule disrupted condition. (**D–F**) Gly- SP induced increases in the influx frequency (**D**), dwell time (**E**), confinement area (**F**) were abrogated by actin disruption but not by microtubule disruption. Values are mean ± s.e. *p<0.01, paired *t*-test. (n = 11 neurons for Gly; n = 9 neurons for Cyto B; n = 9 neurons for Noco, each neuron with at least 10 sPH-AP-QDs).

Next, we tested whether the disruption of actin or microtubules could affect SV dynamics during Gly-SP. We found that actin disruption completely blocked any changes in the dynamics of the SVs by Gly-SP, suggesting that the actin cytoskeleton is required for induction of Gly-SP (Fig. 4). Disruption of the microtubules by nocodazole, however, did not affect Gly-SP induced changes in the dynamics of the SVs (Fig. 4, Fig. S6D).

**Figure 5:**
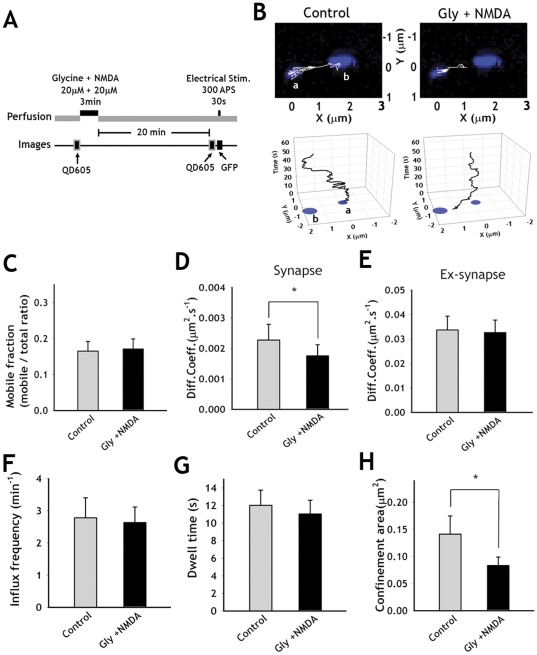
Effects of NMDA-SD on the diffusive behaviors of SVs. (**A**) Schematic diagrams of NMDA-SD induction and imaging protocol. Hippocampal neurons labeled with sPH-AP-QDs were placed in the Mg^2+^-free extracellular solution, and applied with 20 µM of glycine/20 µM NMDA for 3 min to induce SD. (**B**) Reconstruction of a sPH-AP-QD trajectories (white) for 60 s over the functional presynaptic terminal(blue) images along the axons before (left panel) and after (right panel) induction of NMDA-SD. Lower panels: sPH-AP-QD traces over the time in 3-dimensional space. Presynaptic terminals labeled as a and b correspond to those in upper panel, respectively. Graphs were rotated for better representation of SV movement over the time (**C**) Comparison of mobile fraction before and after induction of NMDA-SD. (**D, E**) Comparison of average diffusion coefficients at synapses (**D**) and at extrasynapses (**E**) after NMDA-SD. (**F–H**) Influx frequency (**F**), dwell time (**G**), confinement area (**H**) after NMDA-SD compared with control conditions. Values are mean ± s.e. *p<0.01, paired *t*-test. (n = 10 neurons, each neuron with at least 10 sPH-AP-QDs).

When we induced NMDA-induced synaptic depression (NMDA-SD) (Fig. 5A) [22], the diffusion coefficient at synapses was significantly decreased while the diffusion coefficient at extrasynapses was not affected (Fig. 5B, D, E, Fig. S6D and Table 1). The confinement area at synapses was also significantly decreased, indicating that the SVs move around slowly in a small confined area within synapses (Table 1). There were no changes in the influx frequency or dwell time by NMDA-SD induction (Fig. 5).

Although QDs have been used to study the kiss-and-run mechanism of SVs [11], QDs are released after a single-round of recycling, thus not applicable for diffusion behavioral studies. Additionally, previous reports regarding diffusion behaviors of SVs were on the movement of the vesicle population rather than on an individual vesicle or on the movement of a single vesicle in a very narrow volume [3], [26], [27]. In this study, using the sPH-AP-QD probe, we were able to study previously unobserved multiple diffusive behaviors and the dynamics of individual SVs at synaptic and extrasynaptic compartments. In addition to a significantly high SNR, the ability to acquire both a QD signal and sPH signal with the same probes could potentially provide access to temporal and functional dynamics of SVs.

There is increasing evidence that clusters of SVs in developing and mature neurons can show a degree of extrasynaptic mobility [3], [28], [29] and recent studies indicated that synaptic vesicles are shared between presynaptic boutons, and recycling-pool vesicles are transported between, and used by, multiple release sites [3], [30]. Very recent study reported that mobile SV packets can fuse with membrane at the sites called hot spots where substantial Ca^2+^ influx occurs [31]. Here, using a QD-based presynaptic probe, we showed that there is considerable sharing of SVs between presynaptic boutons, which can be dynamically modulated by synaptic activities. We found that by the induction of Gly-SP, the diffusion coefficient of the SVs increased ∼3-fold and the confinement area increased by 70%. These results indicate that the SVs move more vigorously at synapses, which may imply more active SV recycling and SV trafficking between populations of recycling vesicle pools. Moreover, spontaneous sharing of SVs between boutons is modified in a direction favoring SV incorporation into the synapse. Therefore, we speculate that Gly-SP causes the increase of 1) the SV recycling , 2) SVs transition between vesicle pools, and 3) SVs number at synapses, which could potentially be related to the increase in the presynaptic efficacy.

Although we have provided evidence to show that Gly-SP induces an increase in the presynaptic release probability (Fig. S5), as for NMDA-SD, there is no known presynaptic measure to substantiate our claim in which the protocol used had effects presynaptically. Thus, we are not fully confident that the NMDA-SD protocol really induces synaptic depression in our condition, except for the fact that glycine with NMDA treatment has been used widely to induce LTD-like changes in culture conditions. This could explain, compared to Gly-SP, why the NMDA-SD resulted in rather limited changes in the dynamics of the SVs. Nevertheless, NMDA-SD resulted in the decrease of the diffusion coefficient and confinement area at synapses, which imply that SVs recycle slowly and their movements between vesicle pools were rather limited.

Estimates of the values for the diffusion coefficient may be altered by the steric hindrance of the QDs upon the diffusion of the SVs. In addition, since synapse is a 3D structure, the SV movement we described here may underestimate the movement of the SV in the z- direction. The diffusion coefficient observed in this study, however, was similar to the values found in other studies [26], [32], [33], thus, tagging QDs to SVs does not seem to affect its mobility significantly. The low mobility of the SVs is attributed to vesicle confinement by synaptic proteins such as synapsins or the cytoskeleton [33], [34]. Because current models regard cytoskeletal elements as stable structural elements, the elimination of the cytoskeleton would release the vesicles to freely diffuse. Unlike these predictions, however, our results showed that the elimination of the actin cytoskeleton induces a dramatic reduction in the diffusion coefficient of the SVs at synapses. It also caused an increase in the mean dwell time and a decrease in the confinement area. These results suggest that the actin cytoskeleton is rather a dynamic element by which vesicle movement is affected rather than a stable structural element that restricts vesicle diffusion. The diffusion coefficient at the extrasynaptic areas was affected not by actin disruption but by microtubule disruption. The nature of the interaction among SVs, the axonal cytoskeleton, and/or other proteins such as synapsins at synapses, or at the extrasynapses, and how these interactions are modulated by synaptic activity requires further investigation.

## Materials and Methods

### Ethics statement

Animal experimental procedures were approved by the Institute of Animal Care and Use Committee of Seoul National University, Korea (Approval ID number: SNU-091006-3).

### Plasmid construction

Bir-ER construct and synaptopHluorin were kindly provided by Alice Y Ting at MIT and by James Rothman at Yale, respectively. sPH was amplified by PCR with acceptor peptide (AP) sequence (KKKGPGGLNDIFEAQKIEWH) contained 3′ primer from sPH plasmid template and was inserted into pcDNA 3.0 vector (Invitrogen, Carlsbad, CA).

### Hippocampal neuron culture and transfection

Cultured hippocampal CA3-CA1 neurons are prepared from embryonic day 18 (E-18) fetal Sprague-Dawley rats as described [22]. Cultured neurons were plated on poly-D-lysine coated 18 mm glass coverslips at a density of 250,000 cells/60 mm dish. Cultures were grown in the Neurobasal medium (Invitrogen) supplemented 2% B-27 (Invitrogen), and 0.5 mM L-glutamine. Neurons were transfected at DIV12 using the calcium-phosphate method [22]. Briefly, 6 µg of cDNA and 9.3 µl of 2 M CaCl_2_ were mixed with distilled water to the total volume of 75 µl, and 75 µl of 2× BBS was added to the mixture. Original medium was changed with transfection medium (MEM, 1 mM pyruvate, 0.6% glucose, 2 mM glutamine, and 10 mM HEPES, pH 7.65), cDNA mixture was added to the cells, and incubated in 5% CO_2_ incubator for 90 min. Neurons were washed 2 times with washing medium (MEM, 1 mM pyruvate, 0.6% glucose, 2 mM glutamine, and 10 mM HEPES, pH 7.35) and replaced with the original culture medium.

### Single SV labeling with streptavidin conjugated quantum dots

Neurons were co-transfected with sPH-AP and Bir-ER. Surface sPH-AP was blocked with 100 nM of unlabeled streptavidin for 5 min on ice followed by 3 times washing in the Tyrode's solution (119 mM NaCl, 2.5 mM KCl, 2 mM CaCl_2_, 2 mM MgCl_2_, 25 mM HEPES, 30 mM glucose, pH 7.4). Cells were incubated with 100 pM of streptavidin conjugated QDs 605 (QDs) in 90 mM high KCl solution (31.5 mM NaCl, 90 mM KCl, 2 mM CaCl_2_, 2 mM MgCl_2_, 25 mM HEPES, 30 mM glucose, pH 7.4) for 3 min and washed for 10 min at room temperature. All solutions included 10 µM 6-cyano-7-nitroquinoxaline-2, 3-dione and 50 µM DL-2-amino-5-phosphonovaleiric acid to reduce spontaneous activity and prevent recurrent excitation.

### Image acquisition and data analysis

Images were obtained with an Olympus IX-71 inverted microscope (Olympus, Tokyo, Japan) with a 40×, 1.0 N.A. or a 60×1.45 N.A. oil lens using a EMCCD camera (iXon885; pixel size 8 µm, Andor Technologies, Belfast, Northern Ireland) driven by MetaMorph Imaging software (Universal Imaging Corporation, West Chester, PA). Light from a mercury lamp was shuttered using a VMM1 Uniblitz shutter (Vincent Associates, Rochester, NY). Time lapse images were acquired every 0.5 s and detected using an excitation filter (475AF40; Omega Filters, Brattleboro, VT) and an emission filter (605WB20; Omega Filters). To monitoring the effect of actin cytoskeletons and microtubules on the SV movements, control time-lapse images were acquired, then 3 µg/ml Cytochalasin B (Sigma-Aldrich, St. Louis, MO) or 5 µg/ml Nocodazole (Merck, Whitehouse Station, NJ) were treated for 20 min and time lapse images were collected again at the same field of view. Finally, neurons were stimulated with 300 APs electrically to verify the localization of the presynaptic terminals. Since ∼20% of signals come from surface resident QDs, to make sure we tracked internally endocytosed QDs in the SVs, each tracking was followed by ammonium chloride treatment and only results showing ∼15% increase in the fluorescence signal were included for analysis.

Gly-SP or NMDA-SD was induced by exposing the cells to 200 µM glycine or 20 µM glycine and 20 µM NMDA in the extracellular solution (110 mM NaCl, 2 mM CaCl_2_, 5 mM KCl, 10 mM HEPES, 30 mM glucose, 0.5 µM TTX, 1 µM strychinine, 20 µM bicuculine methiodide, pH 7.4) for 3 min. CNQX and AP-5 are omitted during glycine/NMDA incubation. Cells were then continuously perfused with the same extracellular solution without glycine or glycine and NMDA for 20 min further at room temperature.

### Single particle tracking and quantitative analysis

Single QDs were identified by their blinking behavior, the random alternation between an emitting state and a non-emitting state. Single QD tracking was performed with MetaMorph Imaging software, in which the center of the spot fluorescence was determined using a Gaussian fit. Analysis and fitting were performed with MathCAD2000 (PTC, Needham, MA) and Origin (OriginLab, Northhamton, MA). Values of the mean square displacement (MSD) were calculated from the trajectories applying the relation:
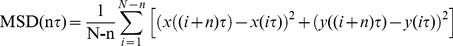
where *xi* and *yi* are the coordinates of an object on frame *i*, *N* is the total number of steps in the trajectory, *τ* is the acquisition time [16]. Diffusion coefficients (*D*) were calculated by fitting the first five points of the MSD curves versus time (*τ*) with the equation MSD(nτ)≈4*Dnτ*. The size of the domain of confinement was estimated by fitting the MSD with the expected generic expression for a confined diffusion [35] as follows:
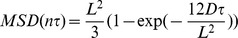
where *L* is the side of a square domain in which diffusion is supposed to be restricted. Single sPH-AP-QD trajectories were reconstructed over the recordings using MetaMorph software. The state of single sPH-AP-QDs was plotted over time. The influx frequency was calculated for each recording by dividing the sum of entries of sPH-AP-QDs into the synapse per minute of detection. Dwell time at synapses was defined as the duration of detection of sPH-AP-QDs at synapses on a recording divided by the number of exits from synapses. Mobile fraction was defined as the number of moving sPH-AP-QDs divided by total number of sPH-AP-QDs labeled during the course of experiment.

### Statistical analysis and image preparation

Statistical analyses were done using SigmaStat (Systat Software) on data compiled and analyzed using Origin and MathCAD2000. Images were prepared for printing using Photoshop and Illustrator (Adobe Systems, San Jose, CA).

### Measurement of QD photoluminescence

To test the pH-dependency of the QDs photoluminescence, hippocampal neurons that expressed biotinylated sPH-APs were labeled with 10 pM QDs and then fixed with 4% paraformaldehyde. Fixed neurons were incubated with Tyrode's solution (pH 5.4) and images were collected 120 pictures with 50 ms exposure time. After image collection, Tyrode's solution/pH 5.4 was change to Tyrode's solution/pH 7.4 and images were collected again. Since its blinking behavior, during acquisition, QD emits either its highest fluorescence, lowest fluorescence, or between. Therefore, maximum intensity means its highest fluorescence intensity and average intensity means averaging value of 120 frames.

For NH_4_Cl treatment, after SVs labeling with 100 pM QDs in 90 mM high KCl Tyrode's solution, single QD fluorescence intensity in neurons was traced for 1 min with 0.5 s interval. Then, 50 mM NH_4_Cl/Tyrode's solution (69 mM NaCl, 50 mM NH_4_Cl, 2.5 mM KCl, 2 mM CaCl_2_, 2 mM MgCl_2_, 25 mM HEPES, 30 mM glucose, pH 7.4) was applied directly to the bath to change the intravesicular pH to pH 7.34.

### Single QD imaging embedded in agarose gel

QDs were diluted to 10 pM in a 1% agarose gel (pH 7.4), then this mixture were mounted in a 18×18 mm square glass coverslip, cooled to room temperature. QDs embedded in an agarose gel were imaged every 0.5 s for 1 min and analyzed their fluorescence intensity using MetaMorph software.

### Transmission electron microscopy

Quantum dot labeled neurons ware fixed with 2% glutaraldehyde in 0.1 M sodium phosphate buffer (PB, pH 7.4) for 1 h at room temperature. To identify the labeled neurons, fixed cells ware observed by fluorescence microscope and scraped around of fluorescence signal positive area. 1% OsO4 and 1.5% potassium ferrocyanide were treated for 1 hr at room temperature and cells were stained by 0.5% uranyl acetate overnight at 4°C and dehydrated in the graded ethanol series. After polymerization in Epon for 2 days at 60°C, the glass coverslips were detached from epon block. Samples were sectioned by EM UC7 (Leica, Wetzlar, Germany) ultramicrotome to 70 nm thickness and contrasted with 0.5% uranyl acetate. TEM images ware collected with a JEM 1400 (Jeol, Tokyo, Japan) electron microscope.

### SynaptopHluorin (sPH) vesicle recycling assay

After labeling the SVs with QDs, coverslips were mounted in a perfusion/stimulation chamber equipped with platinum-iridium field stimulus electrode (EC-S-10, LCI, Seoul, South Korea). Cells were continuously perfused with Tyrode's solution at room temperature. Time lapse images were acquired every 10 s for 5 min. From the fourth frame, cells were stimulated (1 ms, 20–50 V, bipolar) for 30 s at 20 Hz using an A 310 Accupulser current stimulator (World Precision Instrument, Sarasota, Fla). Quantitative measurements of the fluorescence intensity at individual boutons were obtained by averaging a selected area of pixel intensities using MetaMorph software. Net fluorescence changes were obtained by subtracting average of the intensities of the first four frames (*F*_0_) from the intensity of each frame (*F*_t_) for individual boutons. Then they were normalized to the maximum fluorescence intensity (*F*_max_ - *F*_0_) and averaged. The decay of fluorescence was fitted with a single exponential. All fitting procedures were done using individual error bias to weight the fit using SigmaPlot 8.0 (Systat Software, Point Richmond, CA). In some experiments in which fluorescence decay deviated from a single-exponential behavior, we obtained the best-fitting single-exponential function from the early portion of decay.

### FM 4-64 exocytosis assay

After 200 µM glycine treatment in the absence of Mg^2+^ in the extracellular solution for 3 min, neurons were incubated for 1–2 h in the original Neurobasal medium. FM 4-64 was used at a concentrationof 15 µM in the Tyrode's solution. Pools of synaptic vesicles were labeled during electrical stimulation for 60 s at 10 Hz in the presence of FM 4-64. After 10 min of washing in dye-free Tyrode's, images were taken with 5 s interval during the stimulation for 2 min at 10 Hz to unload the FM 4-64. Images were acquired using using a EMCCD camera driven by MetaMorph Imaging software with a FM 4-64 optimized filter set (Omega Optical).

## Supporting Information

Figure S1 Determination of the number of sPH-AP-QDs in a single synaptic vesicle. We have sparsely embedded 10 pM of streptavidin conjugated QD 605 in a 1% agarose gel, selected QDs that show a characteristic blinking behavior, and measured the intensity of their photoluminescence. We have labeled neurons with 100 pM of QD-streptavidins, and measured the intensity of QDs photoluminescence. (**A**) Representative traces of the fluorescence intensity of single QD in an agarose gel (upper graph) and sPH-AP-QDs in the neuron (lower graph). Both show characteristic blinking behaviors. (**B**) The unitary intensity of QDs photoluminescence in an agarose gel closely matched that of sPH-AP-QDs in the neurons indicating a single vesicle contains a single QD (14.03 a.u. for agarose, 12.46 a.u. for synaptic vesicle). (**C**) We have measured the size of the streptavidin conjugated QD 605 used in a current study using transmission electron microscopy (TEM). It is 13.3±1.2 nm (n = 100).(**D**) The localization of sPH-AP-QDs in the synaptic vesicle was established by TEM, which provided the direct evidence that most of synaptic vesicle contained a single sPH-AP-QD (arrows). QDs were never detected intracellularly, indicating that QDs were internalized only through synaptic vesicle endocytosis during the course of the experiments.
(TIF)

Figure S2 sPH-AP-QDs are localized in the synaptic vesicle lumen rather than on the surface. (**A**) Maximum (left) and average (right) intensities of immobilized QDs on the surface of the neurons that express sPH-AP in the pH 5.4 and pH 7.4 Tyrode's solution. We took 120 pictures with 50 ms exposure time. Since its blinking behavior, during acquisition, QD emits either its highest fluorescence, lowest fluorescence, or between. Therefore, maximum intensity means its highest fluorescence intensity and average intensity means averaging value of 121 frames. When pH is changed from 5.4 to 7.4, the fluorescence intensity of QDs showed ∼15% increase on the maximum intensity (131.08±2.51 to 150±4.39) while ∼30% increase on the average intensity (53.90±2.95 to 69.97±4.36). Values are mean ± s.e. *p<0.01, paired *t*-test (n = 60 QDs for all experiments). (**B**) Average intensity profiles from 5 different QDs labeled neurons before and after NH_4_Cl challenge. sPH-AP-QDs fluorescence intensity increased by ∼15% when pH was raised from 5.48 (vesicular) to 7.34 (extracellular) after treatment of 50 mM NH_4_Cl Tyrode's solution. These results indicate that sPH-AP-QDs were harbored within the synaptic vesicles. (**C**) Fluorescence intensities of actively blinking sPH-AP-QDs in the neurons were compared before and after 50 mM of NH_4_Cl treatment. Most of sPH-AP-QDs in a given neuron responded to NH_4_Cl challenge (i.e. fluorescence increase), thus classified as the synaptic vesicular fraction (81.55±6.55%), while sPH-AP-QDs that didn't show any fluorescence change were classified as the surface fraction (18.45±6.55%). Since there is ∼20% of surface resident pool of sPH-biotin, all experiments were done after blocking the surface pool with unlabeled streptavidin (See Methods for details). (**D**) To label QDs on the surface, we have used TTX to block the neuronal activity. We preincubated the neurons with 1 µM of TTX for 20 min, followed by incubation of 20 pM of streptavidin-conjugated QD 605 in the TTX-Tyrode's solution for 5 min at 4°C. After washing with ice-cold Tyrode's solution, neurons were imaged in the TTX- Tyrode's solution. The surface labeling protocol resulted in QDs having diffusion coefficient of 0.00784±0.00028 at synapses and 0.0544±0.0009 at extrasynapses. These values are significantly faster than those of QDs labeled using our labeling protocols (0.00287±0.00032 at synapses; 0.0367±0.0032 at extrasynapses. Our labeling protocol resulted in about 70% of low diffusion coefficient population(0.00287±0.00032, which is in the SV) and 30% of high diffusion coefficient population (0.00837±0.00124, which is on the surface at synapses.
(TIF)

Figure S3 (**A**) The kinetics of synaptic vesicle recycling upon sPH-AP-QDs labeling. We have transfected neurons with sPH-AP and Bir-ER at DIV 12 and labeled them with 1 nM of streptavidin conjugated QD 605 at DIV 17. Judging from their fluorescence intensity, we estimate that upto 30% of total recycling pool can be labeled with streptavidin conjugated QD 605. Average intensity profiles of synaptic boutons expressing sPH, plotted as Δ*F*/*F*0 against time, following stimulation with 600 action potentials at 20 Hz. Filled circle indicates unlabeled neurons (n = 7 neurons) and open triangle indicates streptavidin conjugated QD 605 labeled neurons (n = 7 neurons). Time constant of sPH declining kinetics (τ): 54.46±2.05 for control (n = 247 boutons), 56.35±3.16 for QD-labeled neurons (n = 307 boutons). No significant difference p = 0.35 (**B**) Exocytotic kinetics of unlabeled control and QD-labeled neurons. The rate of exocytosis was obtained from the exponential fit to the data during stimulation ( Time constant (τ): 25.07±1.08 for control, 26.25±3.54 for QD labeled neurons, n = 7 neurons)
(TIF)

Figure S4 (**A**) Representative pictures of microtubules from the control neurons, neurons treated with 5 µg/ml or 10 µg/ml of nocodazole. Lower panels are enlarged pictures after nocodazole treatment at 37°C for 20 min, neurons were fixed in pre-cooled 100% methanol at −20°C for 10 min and blocked with 10% BSA/PBS at 37°C for 20 min. The neurons were incubated with beta-tubulin antibody/3% BSA/PBS at 37°C for 2 hr and incubated with Oregon Green conjugated secondary antibody/3% BSA/PBS at 37°C for 45 min. The bottom panels are enlarged pictures. Scale bars, upper: 25 µm, bottom: 5 µm.(**B**) Comparison of mobile fraction before and after 5 µg/ml or 10 µg/ml of nocodazole treatment. (**C, D**) Comparison of diffusion coefficients averaged during synaptic (**C**) and extrasynaptic (**D**) sequences before and after 5 µg/ml or 10 µg/ml of nocodazole treatment. (**E, F**) Comparison of influx frequency (**E**), dwell time (**F**) before and after 5 µg/ml or 10 µg/ml of nocodazole treatment. Values are mean ± s.e. *p<0.01, paired *t*-test. (n = 9 neurons for 5 µg/ml of nocodazole , n = 7 neurons for 10 µg/ml of nocodazole). We have analyzed ∼10 QDs movements/neuron.
(TIF)

Figure S5 Presynaptic release was enhanced by Gly-SP induction in cultured neurons. (**A**) Effect of Gly-SP on FM 4-64 destaining kinetics which is a reliable measure of release probability. Average intensity profiles of presynaptic boutons loaded with FM 4-64, plotted as Δ*F*/*F*_0_ against time following stimulation with 1200 action potentials at 10 Hz. Trianlges: control, filled-circles (Gly): Gly-SP induction, open-circles (Gly+APV): Gly-SP induction in the presence of APV. (**B**) The decay kinetics of FM4-64 fluorescence were fitted by a single exponential function with time constants (τ = 82.09±4.31 (n = 4) for control; τ = 53.16±1.75 (n = 4) for glycine; τ = 85.54±7.90 (n = 4) for glycine with APV. We have analyzed 3–5 neurons/experiment and each neuron contained more than 20 boutons/neuron. Values are mean ± s.e. *p<0.01, (Anova and Turkey's HSD post hoc test)
(TIF)

Figure S6 Time-dependent MSD plot of sPH-AP-QDs in neuron. (**A**) Time-dependent average MSD values are shown for control (gray circle, n = 50 QDs) and Cytochalasin B treatment (black circle, n = 50 QDs) at synapses. (**B**) Time-dependent average MSD values are shown for control (gray circle, n = 50 QDs) and Nocodazole treatment (black circle, n = 50 QDs) at extrasynapses. (**C**) Time-dependent average MSD values are shown for control (gray circle, n = 50 QDs) and Gly-SP induction without APV (black circle, n = 50 QDs) at syanpses. (**D**) Time-dependent average MSD values are shown for control (gray circle, n = 50 QDs) and Gly-SP induction under nocodazole treatment (black circle, n = 50 QDs) at syanpses. (**E**) Time-dependent average MSD values are shown for control (gray circle, n = 50 QDs) and NMDA-SD induction (black circle, n = 50 QDs) at syanpses.
(TIF)

Discussion S1 (DOC)
