# Supplementary figures and images for: Dynamics of Multiple Trafficking Behaviors of Individual Synaptic Vesicles Revealed by Quantum-Dot Based Presynaptic Probe
Source: PLoS One. 2012 May 29;7(5):e38045. doi: 10.1371/journal.pone.0038045 (PMC3362565; doi:10.1371/journal.pone.0038045)

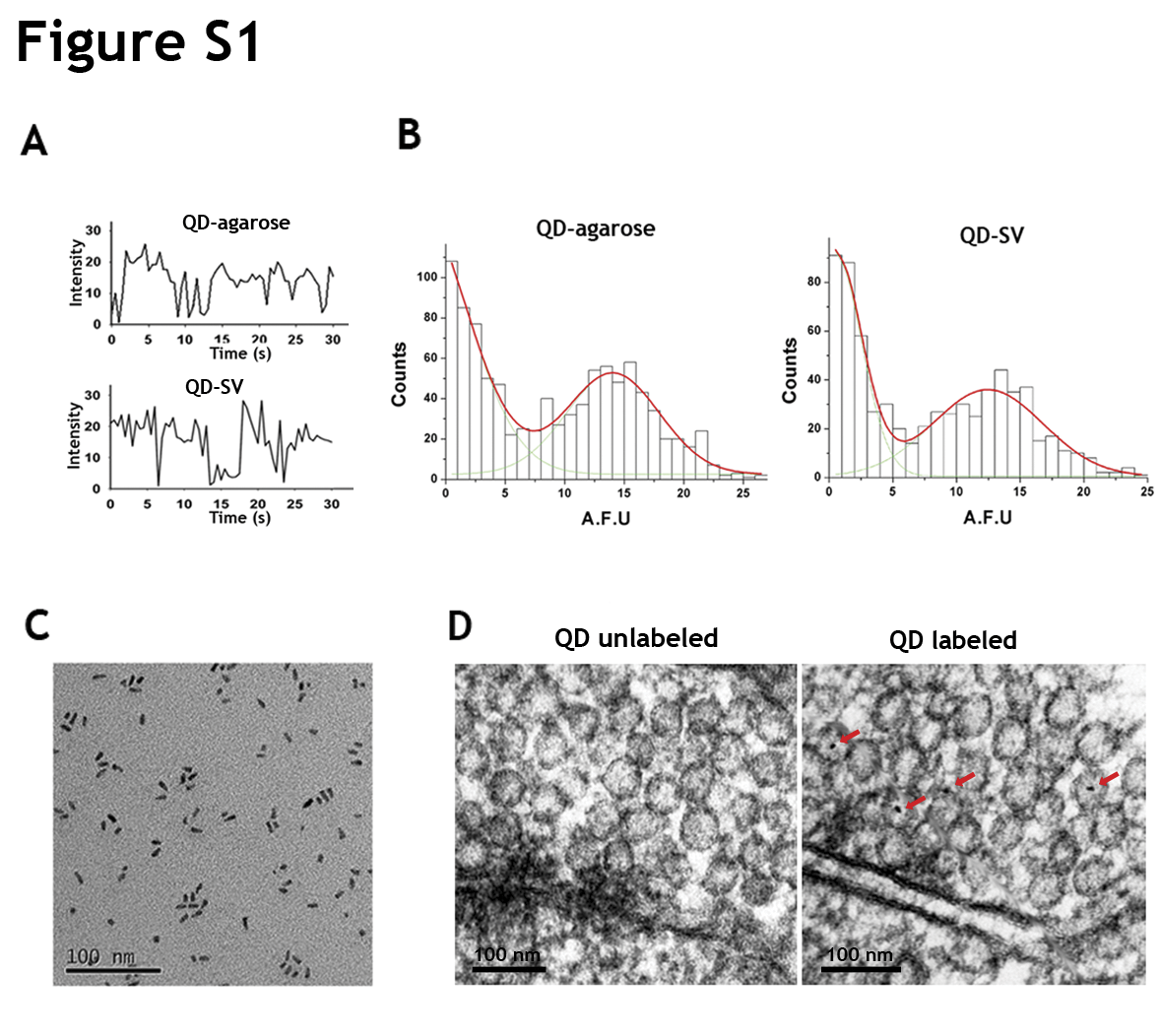

Supplement: Figure S1 — Determination of the number of sPH-AP-QDs in a single synaptic vesicle. We have sparsely embedded 10 pM of streptavidin conjugated QD 605 in a 1% agarose gel, selected QDs that show a characteristic blinking behavior, and measured the intensity of their photoluminescence. We have labeled neurons with 100 pM of QD-streptavidins, and measured the intensity of QDs photoluminescence. (A) Representative traces of the fluorescence intensity of single QD in an agarose gel (upper graph) and sPH-AP-QDs in the neuron (lower graph). Both show characteristic blinking behaviors. (B) The unitary intensity of QDs photoluminescence in an agarose gel closely matched that of sPH-AP-QDs in the neurons indicating a single vesicle contains a single QD (14.03 a.u. for agarose, 12.46 a.u. for synaptic vesicle). (C) We have measured the size of the streptavidin conjugated QD 605 used in a current study using transmission electron microscopy (TEM). It is 13.3±1.2 nm (n = 100).(D) The localization of sPH-AP-QDs in the synaptic vesicle was established by TEM, which provided the direct evidence that most of synaptic vesicle contained a single sPH-AP-QD (arrows). QDs were never detected intracellularly, indicating that QDs were internalized only through synaptic vesicle endocytosis during the course of the experiments. (TIF) [file pone.0038045.s001.tif]

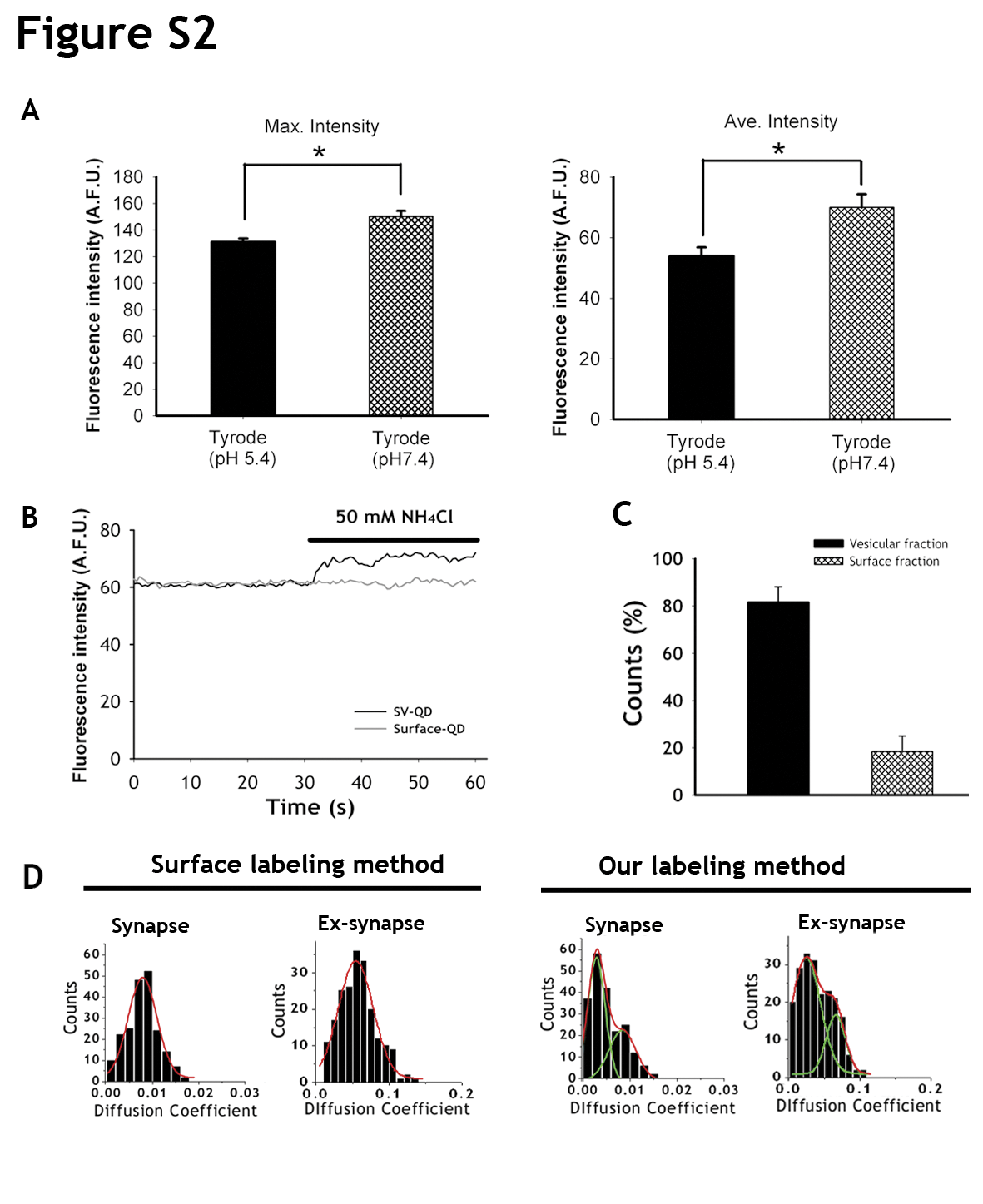

Supplement: Figure S2 — sPH-AP-QDs are localized in the synaptic vesicle lumen rather than on the surface. (A) Maximum (left) and average (right) intensities of immobilized QDs on the surface of the neurons that express sPH-AP in the pH 5.4 and pH 7.4 Tyrode's solution. We took 120 pictures with 50 ms exposure time. Since its blinking behavior, during acquisition, QD emits either its highest fluorescence, lowest fluorescence, or between. Therefore, maximum intensity means its highest fluorescence intensity and average intensity means averaging value of 121 frames. When pH is changed from 5.4 to 7.4, the fluorescence intensity of QDs showed ∼15% increase on the maximum intensity (131.08±2.51 to 150±4.39) while ∼30% increase on the average intensity (53.90±2.95 to 69.97±4.36). Values are mean ± s.e. *p<0.01, paired t-test (n = 60 QDs for all experiments). (B) Average intensity profiles from 5 different QDs labeled neurons before and after NH4Cl challenge. sPH-AP-QDs fluorescence intensity increased by ∼15% when pH was raised from 5.48 (vesicular) to 7.34 (extracellular) after treatment of 50 mM NH4Cl Tyrode's solution. These results indicate that sPH-AP-QDs were harbored within the synaptic vesicles. (C) Fluorescence intensities of actively blinking sPH-AP-QDs in the neurons were compared before and after 50 mM of NH4Cl treatment. Most of sPH-AP-QDs in a given neuron responded to NH4Cl challenge (i.e. fluorescence increase), thus classified as the synaptic vesicular fraction (81.55±6.55%), while sPH-AP-QDs that didn't show any fluorescence change were classified as the surface fraction (18.45±6.55%). Since there is ∼20% of surface resident pool of sPH-biotin, all experiments were done after blocking the surface pool with unlabeled streptavidin (See Methods for details). (D) To label QDs on the surface, we have used TTX to block the neuronal activity. We preincubated the neurons with 1 µM of TTX for 20 min, followed by incubation of 20 pM of streptavidin-conjugated QD 605 in the TTX-Tyrode's [file pone.0038045.s002.tif]

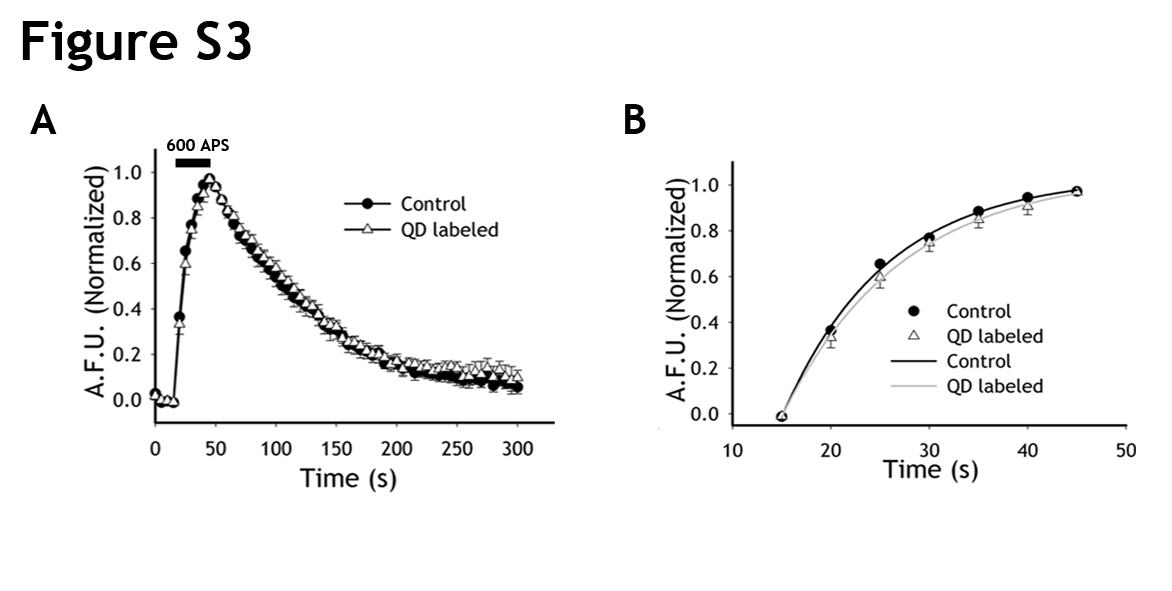

Supplement: Figure S3 — (A) The kinetics of synaptic vesicle recycling upon sPH-AP-QDs labeling. We have transfected neurons with sPH-AP and Bir-ER at DIV 12 and labeled them with 1 nM of streptavidin conjugated QD 605 at DIV 17. Judging from their fluorescence intensity, we estimate that upto 30% of total recycling pool can be labeled with streptavidin conjugated QD 605. Average intensity profiles of synaptic boutons expressing sPH, plotted as ΔF/F0 against time, following stimulation with 600 action potentials at 20 Hz. Filled circle indicates unlabeled neurons (n = 7 neurons) and open triangle indicates streptavidin conjugated QD 605 labeled neurons (n = 7 neurons). Time constant of sPH declining kinetics (τ): 54.46±2.05 for control (n = 247 boutons), 56.35±3.16 for QD-labeled neurons (n = 307 boutons). No significant difference p = 0.35 (B) Exocytotic kinetics of unlabeled control and QD-labeled neurons. The rate of exocytosis was obtained from the exponential fit to the data during stimulation ( Time constant (τ): 25.07±1.08 for control, 26.25±3.54 for QD labeled neurons, n = 7 neurons) (TIF) [file pone.0038045.s003.tif]

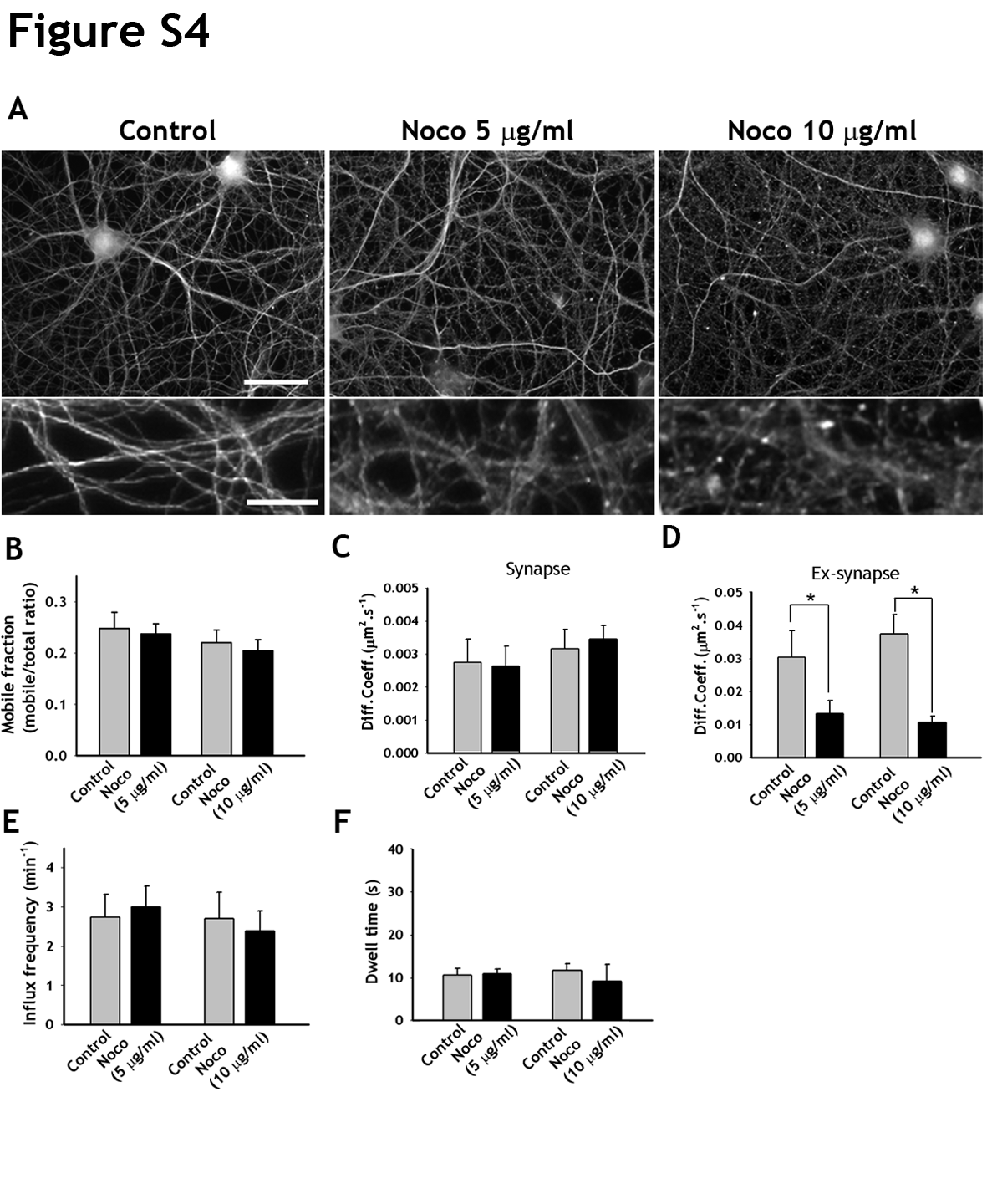

Supplement: Figure S4 — (A) Representative pictures of microtubules from the control neurons, neurons treated with 5 µg/ml or 10 µg/ml of nocodazole. Lower panels are enlarged pictures after nocodazole treatment at 37°C for 20 min, neurons were fixed in pre-cooled 100% methanol at −20°C for 10 min and blocked with 10% BSA/PBS at 37°C for 20 min. The neurons were incubated with beta-tubulin antibody/3% BSA/PBS at 37°C for 2 hr and incubated with Oregon Green conjugated secondary antibody/3% BSA/PBS at 37°C for 45 min. The bottom panels are enlarged pictures. Scale bars, upper: 25 µm, bottom: 5 µm.(B) Comparison of mobile fraction before and after 5 µg/ml or 10 µg/ml of nocodazole treatment. (C, D) Comparison of diffusion coefficients averaged during synaptic (C) and extrasynaptic (D) sequences before and after 5 µg/ml or 10 µg/ml of nocodazole treatment. (E, F) Comparison of influx frequency (E), dwell time (F) before and after 5 µg/ml or 10 µg/ml of nocodazole treatment. Values are mean ± s.e. *p<0.01, paired t-test. (n = 9 neurons for 5 µg/ml of nocodazole , n = 7 neurons for 10 µg/ml of nocodazole). We have analyzed ∼10 QDs movements/neuron. (TIF) [file pone.0038045.s004.tif]

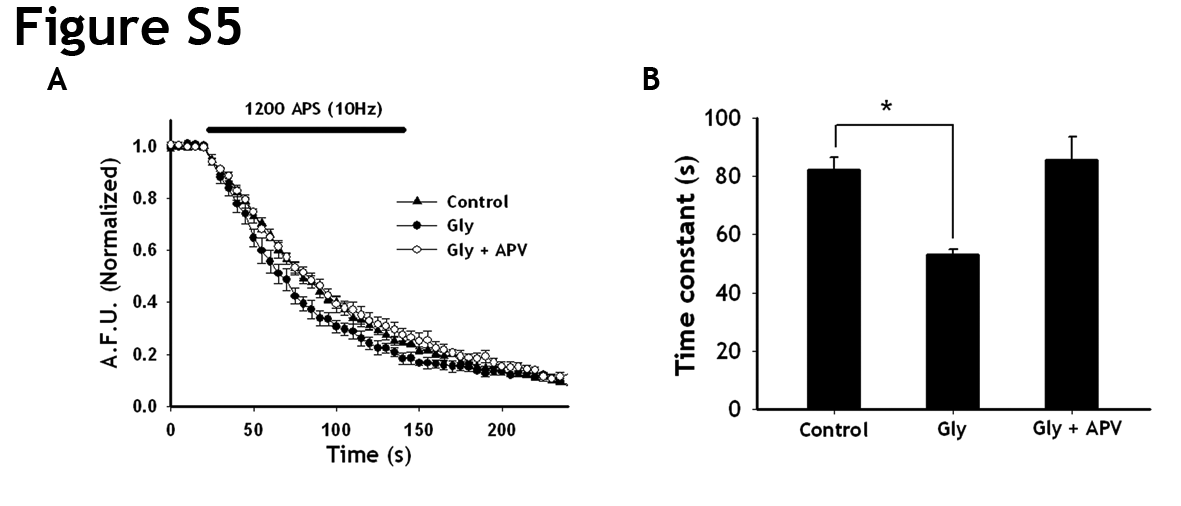

Supplement: Figure S5 — Presynaptic release was enhanced by Gly-SP induction in cultured neurons. (A) Effect of Gly-SP on FM 4-64 destaining kinetics which is a reliable measure of release probability. Average intensity profiles of presynaptic boutons loaded with FM 4-64, plotted as ΔF/F 0 against time following stimulation with 1200 action potentials at 10 Hz. Trianlges: control, filled-circles (Gly): Gly-SP induction, open-circles (Gly+APV): Gly-SP induction in the presence of APV. (B) The decay kinetics of FM4-64 fluorescence were fitted by a single exponential function with time constants (τ = 82.09±4.31 (n = 4) for control; τ = 53.16±1.75 (n = 4) for glycine; τ = 85.54±7.90 (n = 4) for glycine with APV. We have analyzed 3–5 neurons/experiment and each neuron contained more than 20 boutons/neuron. Values are mean ± s.e. *p<0.01, (Anova and Turkey's HSD post hoc test) (TIF) [file pone.0038045.s005.tif]

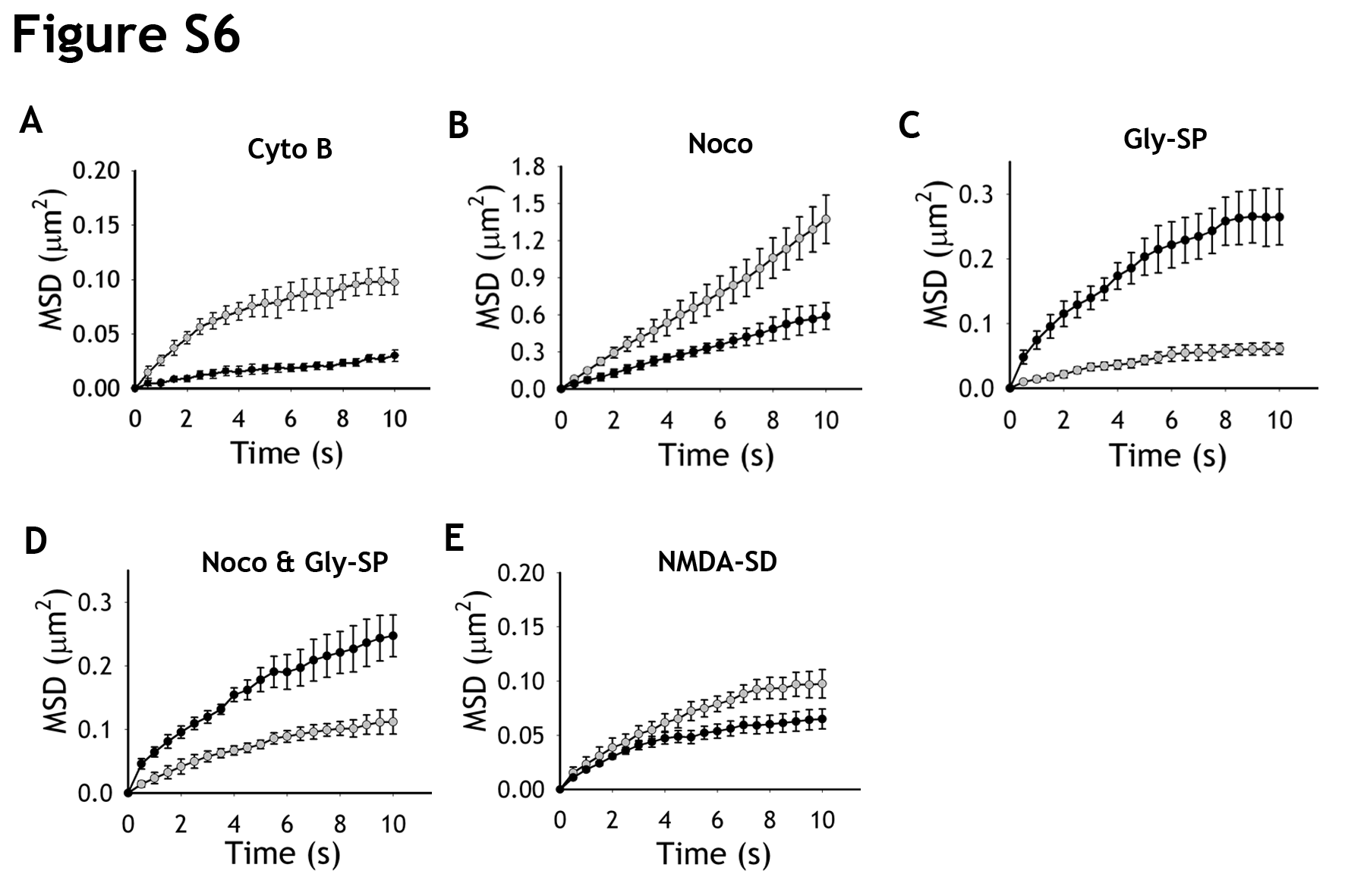

Supplement: Figure S6 — Time-dependent MSD plot of sPH-AP-QDs in neuron. (A) Time-dependent average MSD values are shown for control (gray circle, n = 50 QDs) and Cytochalasin B treatment (black circle, n = 50 QDs) at synapses. (B) Time-dependent average MSD values are shown for control (gray circle, n = 50 QDs) and Nocodazole treatment (black circle, n = 50 QDs) at extrasynapses. (C) Time-dependent average MSD values are shown for control (gray circle, n = 50 QDs) and Gly-SP induction without APV (black circle, n = 50 QDs) at syanpses. (D) Time-dependent average MSD values are shown for control (gray circle, n = 50 QDs) and Gly-SP induction under nocodazole treatment (black circle, n = 50 QDs) at syanpses. (E) Time-dependent average MSD values are shown for control (gray circle, n = 50 QDs) and NMDA-SD induction (black circle, n = 50 QDs) at syanpses. (TIF) [file pone.0038045.s006.tif]
