# Supplementary material for: Dynamics of Multiple Trafficking Behaviors of Individual Synaptic Vesicles Revealed by Quantum-Dot Based Presynaptic Probe
Source: PLoS One. 2012 May 29;7(5):e38045. doi: 10.1371/journal.pone.0038045 (PMC3362565; doi:10.1371/journal.pone.0038045)
Supplement: Discussion S1 — (DOC) [file pone.0038045.s007.doc]

**SUPPLEMENTAL DISCUSSION**

The one of concerns regarding our QD labeling protocols is that there is a possibility in that QDs are in the endosomes rather than in the SVs. Although there is still intense debate as to SVs go through endosome during their recycling or directly recycle without involvement of endosome, we assume if endosome retains QDs during our labeling, it might contain more than a single QDs, thus would show different blinking behaviors. Besides, the previous paper from Goda’s group showed that a group of SVs clusters not endosomes are found to be translocated inter-boutonally, supporting our claims. But still, we must admit that we cannot completely rule out whether QDs are in the endosome from the current study.

Commercially available QDs (e.g. Invitrogen) are known to have dozens of streptavidins conjugated per QD and thus several dozens of binding site to biotin. As such, one QDs may holds to many spH-biotins and thus cause artificial clustering of spH. But since more than 70 copies of VAMP2 are known to be present in a single vesicle and there is yet no known effect of VAMP2 on the SVs translocation, except its effect on the SVs fusion with membrane, we assume that once QDs get inside vesicle, it won’t induce any noticeable changes in the SV trafficking behaviors. Besides, the diffusion coefficient observed in this study was similar to the values found in other studies (Table 1).

Although we used diffusive parameters to describe SV movement, as we discussed in the text, SV movement is way slow compared to the predicted value for free diffusion in aqueous medium. This relatively low mobility of SV has been attributed to vesicle confinement by synaptic proteins such as synapsin or cytoskeletons. Nevertheless, SVs movement at synapses and at extrasynapses shows characteristics that we could use diffusive parameters to describe it. For example, at extrasynapses SVs movement shows free diffusion-like behaviors while it shows low mobility at synapses which can be described as confined-movement when we compared it with that at extrasynapses (Fig.1 D). It, however, doesn’t mean that SV movement is mainly diffusive. Many previous studies showed that SVs are translocated along the axon by microtubules and are recruited into releasing pool by myosin and actin. We also showed that disruption of either actin or microtubule affected SV dynamics, implying the involvement of cytoskeletons. The direction of SV movement was, however, random, thus we didn’t see any directionality that we could define SV movement as either anterograde (to the axonal tip) or retrograde (to the soma) at least during the period of observation. Both forward and backward movements have similar diffusion coefficients. SVs might be dynamically attached or detached to cytoskeletons and diffuse in small cages located near the membrane. It certainly requires further intensive study.

**REFERENCES**

1. Darcy KJ, Staras K, Collinson LM, Goda Y (2006) Constitutive sharing of recycling synaptic vesicles between presynaptic boutons. Nat Neurosci 9: 315-321.

2. Staras K, Branco T, Burden JJ, Pozo K, Darcy K, et al. (2010) A vesicle superpool spans multiple presynaptic terminals in hippocampal neurons. Neuron 66: 37-44.

3. McGuinness TL, Brady ST, Gruner JA, Sugimori M, Llinas R, et al. (1989) Phosphorylation-dependent inhibition by synapsin I of organelle movement in squid axoplasm. J Neurosci 9: 4138-4149.

4. Yeung C, Shtrahman M, Wu XL (2007) Stick-and-diffuse and caged diffusion: a comparison of two models of synaptic vesicle dynamics. Biophys J 92: 2271-2280.
